# Universal kinetics of imperfect reactions in confinement

**DOI:** 10.1038/s42004-021-00591-2

**Published:** 2021-11-11

**Authors:** Thomas Guérin, Maxim Dolgushev, Olivier Bénichou, Raphaël Voituriez

**Affiliations:** 1 Laboratoire Ondes et Matière d’Aquitaine, CNRS/University of Bordeaux, F-33400 Talence, France; 2 Laboratoire de Physique Théorique de la Matière Condensée, CNRS/Sorbonne University, 4 Place Jussieu, 75005 Paris, France; 3 Laboratoire Jean Perrin, CNRS/Sorbonne University, 4 Place Jussieu, 75005 Paris, France

**Keywords:** Chemical physics, Reaction kinetics and dynamics, Structural properties, Reaction mechanisms

## Abstract

Chemical reactions generically require that particles come into contact. In practice, reaction is often imperfect and can necessitate multiple random encounters between reactants. In confined geometries, despite notable recent advances, there is to date no general analytical treatment of such imperfect transport-limited reaction kinetics. Here, we determine the kinetics of imperfect reactions in confining domains for any diffusive or anomalously diffusive Markovian transport process, and for different models of imperfect reactivity. We show that the full distribution of reaction times is obtained in the large confining volume limit from the knowledge of the mean reaction time only, which we determine explicitly. This distribution for imperfect reactions is found to be identical to that of perfect reactions upon an appropriate rescaling of parameters, which highlights the robustness of our results. Strikingly, this holds true even in the regime of low reactivity where the mean reaction time is independent of the transport process, and can lead to large fluctuations of the reaction time - even in simple reaction schemes. We illustrate our results for normal diffusion in domains of generic shape, and for anomalous diffusion in complex environments, where our predictions are confirmed by numerical simulations.

## Abstract (web summary)

Chemical reactions often require multiple random encounters between reactants, but a general, analytical treatment such imperfect transport-limited/influenced reactions in confined spaces has not yet been proposed. Here, the authors predict the full kinetics of these reactions for Markovian processes in large confining volumes.

## Introduction

The First Passage Time (FPT) quantifies the time needed for a random walker to reach a target site^1–10^. This observable is involved in various areas of biological and soft matter physics and is particularly relevant in the context of reaction kinetics, because two reactants have to meet before any reaction can occur^11–13^. When the reaction is *perfect*, i.e. when it occurs for certain upon the first encounter, its kinetics is controlled by the first passage statistics of one reactant, described as a random walker, to a target site. Of note, earlier works have determined the mean^2,8,14^ and the full asymptotic distribution^15,16^ of first passage times in confinement for broad classes of transport processes.

While most of the literature focuses on perfect reactions, the case of *imperfect* reactions (i.e. which do not occur with certainty upon the first encounter) arises in a variety of contexts (see^17^ for a recent review), if a reaction occurs only when reactants meet with prescribed orientations^12^ or after crossing an energy^18^ (or entropy^19^) activation barrier, if the target site is only partially covered by reactive patches^20^, or in the case of gated reactions where the target (or the reactant) switches between reactive and inactive states^21,22^.

The formalism to calculate the rate of imperfect reactions between diffusive spherical particles in the dilute regime (thus in infinite space) is well established^12,23–25^. However, geometric confinement has proved to play an important role in various contexts, such as reactions in microfabricated reactors or in cellular compartments. Yet, the kinetics of imperfect reactions in a confined volume is still only partially understood: existing methods are restricted to (i) diffusive (or amenable to diffusive) transport processes^26–29^ and most of the time (ii) specific shapes of confining volume^30–33^ (spherical or cylindrical). In fact, a general theoretical framework to quantify the kinetics of imperfect reactions involving non-Brownian transport (such as anomalous diffusion in complex environments^34^) in general confined domains is still missing.

**Fig. 1:**
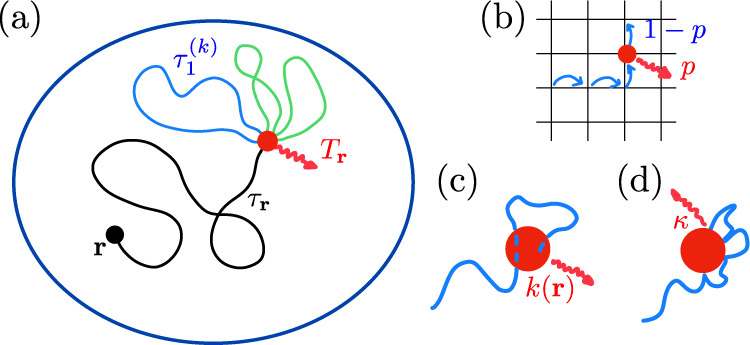
Imperfect reaction kinetics in confinement. (**a**) In the case of imperfect reactions, multiple random interaction events between reactants are typically required before reaction occurs. The reaction time *T*_**r**_ for a random walker starting from **r** with a target (red dot) can then be written $T_{{{{{{\bf{r}}}}}}}= \tau_{{{{{{{\bf{r}}}}}}}} + \mathop{\sum}\nolimits_{k=2}^{n} \tau^{(k)}_1$, where *τ*_**r**_ is the first passage time (FPT) to the target, *n* is the total number of visits to the target before reaction, and ${\tau }_{1}^{(k)}$ is a first return time to the target. (**b**) In the case of discrete space models, imperfect reactivity is parametrized by the probability *p* that reaction occurs at each visit of the random walker to the target. In the case of continuous space models, imperfect reactivity is modeled either by (**c**) a reaction rate *k*(**r**) when the random walker is within the reactive volume that defines the target, or (**d**) partially absorbing boundary conditions (parametrized by *κ*) at the target boundary.

Here, we propose a formalism that determines the full kinetics of imperfect reactions in confinement for general Markovian processes in the large confining volume limit (see Fig. 1). This allows us to answer the following questions: (i) Is reaction limited by transport or reactivity? (ii) What is the magnitude of the fluctuations of the reaction time? In particular, is the first moment sufficient to fully determine reaction kinetics? (iii) Do reaction kinetics depend on the choice of model of imperfect reactivity– namely partially reflecting (Robin) conditions^23,35,36^ or sink with locally uniform absorption rate^24,37^ in continuous models, or finite reaction probability in discrete models?

## Results and discussion

### Discrete model of imperfect reactions

A first straightforward definition of imperfect reactivity is based on the statistics of encounter events between reactants, and thus requires a discrete description of the dynamics. We therefore start by considering a Markovian random walker moving on a discrete space (or network) of *N* sites. We consider a continuous-time dynamics with exponentially distributed waiting times on each site, where *ν*_*i*_ denotes the jump rate from site *i* to any neighboring site. The reactive site is denoted *i* = 0. Imperfect reactivity is then naturally defined as follows: each time the walker visits the reactive site, the reaction occurs with probability *p*, and the random walk continues without reaction with probability 1 − *p*. We call *T*_**r**_(*p*) the *reaction time* and *F*(*T*∣**r**, *p*) its probability density function (PDF) for a random walker starting from **r**. Next, we call *τ*_**r**_ the *first passage time* to the reactive site starting from ***r*** (including the residence time on the reactive site), and we call F^*^(*τ*_**r**_∣**r**) its PDF. We also introduce the *first return time* to the target *τ*_1_ (i.e., the first passage time to the target starting from any site at distance 1 from the target) and ${F}_{1}^{* }({\tau }_{1})$ its PDF. The probability that a reaction happens after exactly *n* visits to the target is given by *p*(1 − *p*)^*n*−1^, in which case *T*_**r**_(*p*) is the sum of the first passage time (starting from **r**) and of *n* − 1 independently distributed first return times (see Fig. 1). Hence, partitioning over the number of visits *n* yields

1 $$F(T| {{{{{{{\bf{r}}}}}}}},p)= \mathop{\sum }\limits_{n=1}^{\infty }\int_{0}^{\infty }{{{{{{{\rm{d}}}}}}}}{\tau }_{{{{{{{{\bf{r}}}}}}}}}\left[\mathop{\prod }\limits_{k=2}^{n}\int_{0}^{\infty }d{\tau }_{1}^{(k)}{F}_{1}^{* }\left({\tau }_{1}^{(k)}\right)\right]\\ \times p{(1-p)}^{n-1}{F}^{* }({\tau }_{{{{{{{{\bf{r}}}}}}}}}| {{{{{{{\bf{r}}}}}}}})\delta \left(T-{\tau }_{{{{{{{{\bf{r}}}}}}}}}-\mathop{\sum }\limits_{k=2}^{n}{\tau }_{1}^{(k)}\right),$$

where ${\tau }_{1}^{(k)}$ represents the return time after *k* − 1 visits to the reactive site. This exact equation is conveniently rewritten after Laplace transform (denoted $\tilde{f}(s)=\int\nolimits_{0}^{\infty }dtf(t){e}^{-st}$ for any function *f*):

2 $$\tilde{F}(s| {{{{{{{\bf{r}}}}}}}},p)=\frac{p\,{\tilde{F}}^{* }(s| {{{{{{{\bf{r}}}}}}}})}{1-(1-p){\tilde{F}}_{1}^{* }(s)}.$$

(see Supplementary Note 1 for details). In the small *s* limit, the property $\tilde{F}(s| {{{{{{{\bf{r}}}}}}}},p)\simeq 1-s\langle {T}_{{{{{{{{\bf{r}}}}}}}}}(p)\rangle$ can be used to obtain an exact expression of the mean reaction time as a function of the mean first passage and the mean return time:

3 $$\langle {T}_{{{{{{{{\bf{r}}}}}}}}}(p)\rangle =\langle {\tau }_{{{{{{{{\bf{r}}}}}}}}}\rangle +\frac{1-p}{p}\langle {\tau }_{1}\rangle .$$

Of note, expression (3) [as well as (2)] is a straightforward consequence of well-known results on random sums^38^, bearing here a clear interpretation because (1 − *p*)/*p* is the mean number of encounter events. Below, we make this result fully explicit by determining 〈*τ*_**r**_〉 and 〈*τ*_1_〉.

The mean return time 〈*τ*_1_〉 can be obtained exactly from the knowledge of the stationary probability density *q*_*i*_ for the random walker to be at site *i* in absence of target; this exact result is known as Kac theorem^39^ and yields

4 $$\langle {\tau }_{1}\rangle =\frac{1}{{q}_{0}{\nu }_{0}}=\frac{N}{{\nu }_{0}},$$

where we have chosen a uniform stationary distribution *q*_*i*_ = 1/*N*, which is realized when the waiting time 1/*ν*_*i*_ at each site is inversely proportional to the number of neighbors^40^.

To gain explicit insight of the behavior of the first reaction times, we next determine 〈*τ*_**r**_〉 and make use of the scale invariance property observed for a broad class of random walks, for which one can define a fractal space dimension *d*_*f*_ (defined such that the characteristic size *R* grows as $R\propto {N}^{1/{d}_{f}}$) and a walk dimension *d*_*w*_ such that the mean square displacement of a random walker scales as $\langle {r}^{2}(t)\rangle \propto {t}^{2/{d}_{w}}$ (without absorption, in unconfined space). Here, we make use of the chemical distance *r*, defined as the minimal number of links between two sites. The first passage kinetics is known to strongly differ for compact walks (*d*_*w*_ > *d*_*f*_, for which the random walker explores densely its surrounding space and the probability to visit a site in infinite space is one) or noncompact walks (*d*_*w*_ < *d*_*f*_, for which an infinite trajectory typically leaves a fraction of unvisited sites which is almost surely one). We shall prove here that the effect of imperfect reactivity is markedly different in these two cases as well.

We first focus on the compact case *d*_*w*_ > *d*_*f*_, for which it was shown^41^ that $\langle {\tau }_{r}\rangle \simeq \alpha N{r}^{{d}_{w}-{d}_{f}}$ for large *r* and large volume *N*, where *α* is a constant independent of *N* and *r*. Following^41^, we assume that this scaling relation holds up even for *r* = 1. Making use of the above determination of 〈*τ*_1_〉, this yields *α* = 1/*ν*_0_. This leads to the following fully explicit determination of the mean reaction time:

5 $$\langle {T}_{r}(p)\rangle \simeq \frac{N\,{r}^{{d}_{w}-{d}_{f}}}{{\nu }_{0}}+\frac{N(1-p)}{p\,{\nu }_{0}}.$$

As expected, the reaction time is thus the sum of a diffusion-controlled (DC) time 〈*τ*_*r*_〉, obtained when *p* = 1, corresponding to the time needed for the reactants to meet, and a reaction controlled (RC) time 〈*τ*_1_〉(1 − *p*)/*p*, which dominates when *p* → 0, corresponding to the sequence of returns to the target needed for the reaction to occur. These two times are equal when *r* ≃ *l*_*c*_, where the characteristic distance *l*_*c*_ is given by

6 $${l}_{c}={[(1-p)/p]}^{1/({d}_{w}-{d}_{f})}.$$

**Fig. 2:**
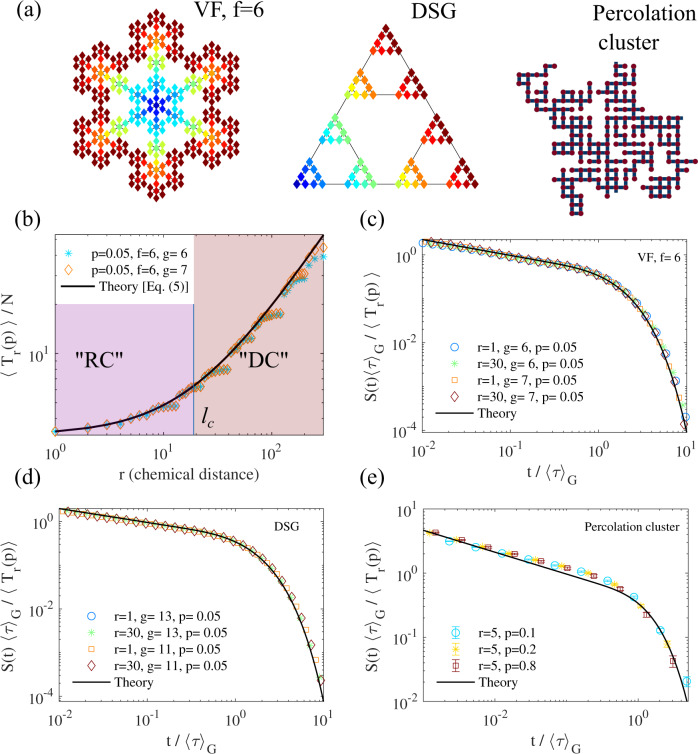
Distributions of reaction times for compact transport. (**a**) Fractal networks for which we performed simulations: Vicsek fractal (VF), here of functionality *f* = 6, dual Sierpinski gasket (DSG), and two-dimensional percolation cluster (a 2D network in which half the bonds are randomly suppressed). For the VF and DSG, the color codes the distance to the reactive site, taken at an apex for DSG and at the center for VFs. (**b**) Mean Reaction time for VF and an absorption probability *p* = 0.05. Reaction Controlled (RC) and Diffusion-controlled (DC) regimes appear respectively below and above the length *l*_*c*_ defined in Eq. (6). (**c**)–(**e**) Survival probabilities, in rescaled coordinates for various generations *g* and initial distances *r* for (**c**): VF, *f* = 6, (**d**): DSG and (**e**): percolation cluster extracted from a 200 × 200 two dimensional square lattice. Details of numerical procedures and additional examples of fractals can be found in Supplementary Note 3, Supplementary Figs. 1–2, and Supplementary Table 1. In (**d**) the error bars represent 95% confidence intervals.

For this compact case, we can therefore split the confining domain into a region where the reaction time is reaction controlled (RC, for *r* < *l*_*c*_), and another one where it is diffusion controlled (DC, *r* > *l*_*c*_), see Fig. 2(b). Remarkably, we note that DC region disappears only when the size *R* of the confining volume becomes of the order of *l*_*c*_, i.e., when $p\ll 1/{R}^{{d}_{w}-{d}_{f}}$; this means that even for very small values of the intrinsic reactivity there will exist DC regions for large enough volumes.

To quantify reaction kinetics at all timescales, the full distribution of the reaction time, or equivalently the survival probability *S*(*t*∣**r**, *p*), defined as the fraction of walkers that have not reacted up to time *t*, is needed. We show in Supplementary Note 2 how to determine *S*(*t*) by evaluating the leading order behavior of all the moments $\langle {T}_{r}^{n}(p)\rangle$ in the large volume limit (defined by *N* → *∞* with all other parameters fixed). Using the additional hypothesis that the scaling behavior of of all moments $\langle {\tau }_{r}^{n}\rangle \sim {r}^{{d}_{w}-{d}_{f}}{R}^{{d}_{f}+(n-1){d}_{w}}$ holds up to *r* = 1, this leads to an explicit determination of the survival probability

7 $$S(t| {{{{{{{\bf{r}}}}}}}},p)\simeq \frac{\langle {T}_{r}(p)\rangle }{{\langle \tau \rangle }_{{{{{{{\mathrm{G}}}}}}}}}\,{{{\Phi }}}_{\nu }\left(\frac{t}{{\langle \tau \rangle }_{{{{{{{\mathrm{G}}}}}}}}}\right),$$

where 〈*τ*〉_G_ is the global mean first passage time, i.e., the average of 〈*τ*_**r**_〉 overall starting positions of the random walker and is independent of *p*. Here, Φ_*ν*_ is a universal function depending only on *ν* = *d*_*f*_/*d*_*w*_, which was obtained^15^ for the first passage problem by relying in the O’Shaughnessy-Procaccia operator^42^ (which is known to provide accurate expressions for propagators for not-too-large distances^43^):

8 $${{{\Phi }}}_{\nu }(\theta )=\mathop{\sum }\limits_{k=0}^{\infty }\frac{{J}_{\nu }({\alpha }_{k}){\alpha }_{k}^{1-2\nu }{{\Gamma }}(\nu ){2}^{2\nu }{\nu }^{2}}{{J}_{1-\nu }({\alpha }_{k}){{\Gamma }}(2-\nu )(1+\nu )}{e}^{-\frac{{\alpha }_{k}^{2}\nu }{2(1-{\nu }^{2})}\theta }.$$

Here Γ is the gamma function, *J* is the Bessel function of the first kind, and *α*_0_ < *α*_1_ < . . . are the zeros of the function *J*_−*ν*_.

Several comments are in order. (i) This main result shows that the functional form of the survival probability is exactly the same as that of the first passage time to the target (obtained for *p* = 1), with a rescaled prefactor 〈*T*_*r*_(*p*)〉 that encompasses all the dependence on the reactivity parameter *p*. It generalizes the result obtained for perfect reactions^15^. (ii) Importantly, the full distribution can be obtained from the knowledge of the first moment 〈*T*_*r*_(*p*)〉 *only*, which makes the mean the key quantity to determine reaction kinetics. (iii) Remarkably, the shape of the reaction time distribution is the same as that of first passage times even in regions of the domain where the mean reaction time is reaction controlled and seemingly independent of the dynamics. As stressed above, the dependence on *p* lies only in the prefactor of the survival probability. This implies that the property of broadly distributed reaction times (nonexponential), characteristic of first passage times for compact transport, is maintained *even for low intrinsic reactivity* in large networks. (iv) The importance of fluctuations can be quantified by the ratio $\langle {T}^{2}\rangle /{\langle T\rangle }^{2} \sim {R}^{{d}_{w}}/\langle T\rangle \gg 1$, which is large in the large volume limit that we consider.

In order to test these predictions for compact processes, we have performed numerical calculations on different examples of both disordered and deterministic fractal networks: the 2-dimensional critical percolation cluster, the Vicsek fractals and the dual Sierpinski gasket, see Fig. 2(a). This enables us to test different values of *d*_*f*_, *d*_*w*_. This class of models has been used to describe transport in disordered media for example in the case of anomalous diffusion in crowded environments like biological cells^44–46^ as a first step to account for geometrical obstruction and binding effects involved in real crowded environments. Our calculations of the reaction times in the case of deterministic fractals are based on a recursive construction of the eigenvalues and eigenfunctions of the connectivity matrix (see Supplementary Note 3 for details) and enable us to obtain exact forms for the Laplace transform of *S*(*t*) for volumes up to *N* ~ 10^6^ sites. As seen on Fig. 2, these numerical results confirm our predictions for the evaluation of the mean first passage time and the rescaled form of the survival probability. These results indicate that our approximations (i.e. the use of the O’Shaughnessy-Procaccia operator, the hypothesis that scaling of all moments hold up to *r* = 1, large volume limit) lead to accurate predictions for the mean reaction time and its full distribution. Of note, even for small values of the reaction probability (*p* = 0.05) the shape of reaction time distribution is exactly the same as that of first passage times, as we predict. In the limit of small *p* (at fixed volume), one expects that the reaction becomes much slower than the transport step, with an exponentially distributed reaction time. However, this exponential regime appears when the length *l*_*c*_ in Eq. (6) becomes comparable to the size *R* of the fractal, i.e. when $p\ll {p}^{* }\equiv 1/{N}^{{d}_{w}/{d}_{f}-1}$. Since p^*^ vanishes for large *N*, this means that the distribution of first passage times remains broadly distributed, with no well-defined reaction rate even for very low values of *p*.

### Noncompact case

We now focus on noncompact processes (*d*_*w*_ < *d*_*f*_). In this case, we make use of the asymptotic FPT distribution, which can be written^15^ as

9 $${F}^{* }(t| r)=\left(1-\frac{\langle {\tau }_{r}\rangle }{{\langle \tau \rangle }_{{{{{{\mathrm{G}}}}}}}}\right)\delta (t)+\frac{\langle {\tau }_{r}\rangle }{{\langle \tau \rangle }_{{{{{{\mathrm{G}}}}}}\,}^{2}}{e}^{-t/{\langle \tau \rangle }_{{{{{{\mathrm{G}}}}}}}},$$

where 〈*τ*〉_G_ has been defined above. The term *δ*(*t*) accounts for the FPT density restricted to trajectories that do not reach the boundary before finding the target, the shape of the function approximated by this *δ*-function does not modify the value of the moments of the distribution in the large volume limit. Now, we make use of this separation of timescales in the FPT distribution and obtain finally the distribution of the reaction time by (i) taking the Laplace transform of (9), (ii) inserting the result into (2) and (iii) taking the inverse Laplace transform. The result of this procedure for the survival probability is

10 $$S(t| r,p)=\frac{\langle {T}_{r}(p)\rangle }{{\langle T(p)\rangle }_{{{{{{{\mathrm{G}}}}}}}}}{e}^{-t/{\langle T(p)\rangle }_{{{{{{{\mathrm{G}}}}}}}}},$$

where the mean reaction time 〈*T*_*r*_(*p*)〉 is deduced from Eqs. (3),(4), and 〈*T*(*p*)〉_G_ = 〈*T*_*r*=*∞*_〉 is the global (indexed by G) mean reaction time, i-e averaged over all starting positions. This result has important consequences. (a) Similarly to the compact case, the shape of the survival probability for imperfect reactions is the same as that of first passage times, with renormalized parameters; in particular the mean gives access to the full distribution and is thus the key quantity to quantify reaction kinetics, as in the compact case. (b) Because the mean FPT scales as 〈*τ*〉 ~ (*N*/*ν*_0_)*g*(*r*), where *g* is a bounded function of *r*, the mean reaction time is dominated by the reaction limited step in the full domain as soon as *p* ≪ 1 for any domain size, in contrast to the compact case. (c) Note that, in Eq. (10) one has *S*(*t* → 0) < 1, which means that it does not take into account the events whose duration does not scale with *R*; the survival probability for these events was identified to the survival probability in infinite space^15,32^. Nevertheless, Eq. (10) can be used to calculate *all the moments* $\langle {T}_{r}^{n}(p)\rangle$ with *n* ≥ 1 in the large volume limit.

### Continuous models: imperfect extended targets

We now aim at discussing alternative microscopic models of imperfect reactivity, which are naturally defined in continuous space. We consider a *d*-dimensional Brownian diffusive particle of diffusion coefficient *D* and analyze two classical models of imperfect targets (see Fig. 1) : (i) a sink region *V*_*r*_ (in which the reaction happens with rate *k*(**r**) and vanishes elsewhere) and (ii) a target region *S*_*r*_ with partially reactive impenetrable boundary. Our above results for discrete models show that the full distribution of reaction times can be obtained in the large volume limit from the first moment of the reaction time only; we conjecture and verify numerically that this holds also for continuous models. We are thus back to determining the mean reaction time in both cases (i) and (ii). In case (i), the mean reaction time 〈*T*(**r**)〉 starting from the position **r** satisfies the following backward equation^47^:

11 $$[D{{{\Delta }}}_{{{{{{{{\bf{r}}}}}}}}}-k({{{{{{{\bf{r}}}}}}}})]\langle T({{{{{{{\bf{r}}}}}}}})\rangle =-1.$$

We next define ${{\Phi }}({{{{{{{\bf{r}}}}}}}})=\mathop{\lim}\nolimits_{V\to \infty }\langle T({{{{{{{\bf{r}}}}}}}})\rangle /V$, and obtain from (11) (and (11) integrated over the volume) :

12 $$[D{{{\Delta }}}_{{{{{{{{\bf{r}}}}}}}}}-k({{{{{{{\bf{r}}}}}}}})]{{\Phi }}({{{{{{{\bf{r}}}}}}}})=0,\,\int d{{{{{{{\bf{r}}}}}}}}\,k({{{{{{{\bf{r}}}}}}}})\,{{\Phi }}({{{{{{{\bf{r}}}}}}}})=1,$$

which fully determines Φ for all ${{{{{{{\bf{r}}}}}}}}\in {{\mathbb{R}}}^{d}$. This formalism can be adapted to the case (ii) of partially reactive target of surface *S*_*r*_ characterized by a surface reactivity *κ* that interpolates from perfect reaction (*κ* → *∞*) to complete absence of reaction (*κ* → 0)^17,23,35,36^; we obtain

13 $${{{\Delta }}}_{{{{{{{{\bf{r}}}}}}}}}{{\Phi }}{| }_{{{{{{{{\bf{r}}}}}}}}\in {{\mathbb{R}}}^{d}\backslash {S}_{r}}=0,\,D{\partial }_{n}{{\Phi }}=\kappa {{\Phi }}{| }_{{{{{{{{\bf{r}}}}}}}}\in {S}_{r}},\,{\int}_{{S}_{r}}dS\kappa {{\Phi }}=1.$$

These equations (12) and (13) generalize the formalism of Ref. ^48^ to the case of imperfect reactions. Note that (i) they can be extended to general Markovian transport operators, for both compact and noncompact cases, and (ii) they are valid for any shape of the confining volume.

To illustrate our formalism, we give solutions for diffusive transport for dimensions *d* = 1, 2, 3 for a spherical target of radius *a*. For the case (i) of a sink region *k*(*r*) = *kθ*(*a* − *r*) with *θ* the Heaviside step function, the MRT outside the sink region (*r* > *a*) reads

14 $$\frac{\langle T\rangle }{V}=\frac{1}{D}\left\{\begin{array}{ll}\frac{r}{2}+\frac{a}{2}\left[\frac{\cosh (\sqrt{K})}{\sqrt{K}\sinh (\sqrt{K})}-1\right]&(d=1)\\ \frac{1}{2\pi }{{{{{{\mathrm{ln}}}}}}}\,\frac{r}{a}+\frac{{I}_{0}\left(\sqrt{K}\right)}{2\pi \sqrt{K}{I}_{1}\left(\sqrt{K}\right)}&(d=2)\\ -\frac{1}{4\pi r}+\frac{\sqrt{K}}{4\pi a[\sqrt{K}-\tanh (\sqrt{K})]}&(d=3)\end{array}\right.$$

where *K* = *ka*^2^/*D* and *I*_0_, *I*_1_ are modified Bessel functions of the first kind. In the case (ii) of a partially reactive impenetrable spherical target we obtain

15 $$\frac{\langle T\rangle }{V}=\left\{\begin{array}{ll}\frac{1}{2D}(r-a)+\frac{1}{2\kappa }&(d=1)\\ \frac{1}{2\pi D}{{{{{{\mathrm{ln}}}}}}}\,(r/a)+\frac{1}{2\pi a\kappa }&(d=2)\\ \frac{1}{4\pi Da}-\frac{1}{4\pi Dr}+\frac{1}{4\pi {a}^{2}\kappa }&(d=3)\end{array}\right.$$

Of note, in *d* = 3, the MRT at *r* = *∞* in Eqs. (15), (14)) is the inverse of effective reactions rates calculated in^23,24^, and in fact the expression (10) then corresponds to the survival probability at long times for *r*/*a* ≫ 1 identified in Ref. ^26^. Finally, our results show that both models are equivalent in the low reactivity limit upon the identification *κS*_*r*_ = *kV*_*r*_; however, in the high reactivity limit, the RC timescales as *κ*^−1^ for surface reactivity, while for sink absorption the RC time displays a non-trivial scaling ∝ *k*^−1/2^, due to the fact that most reaction events occur in a small penetration length from the target surface.

**Fig. 3:**
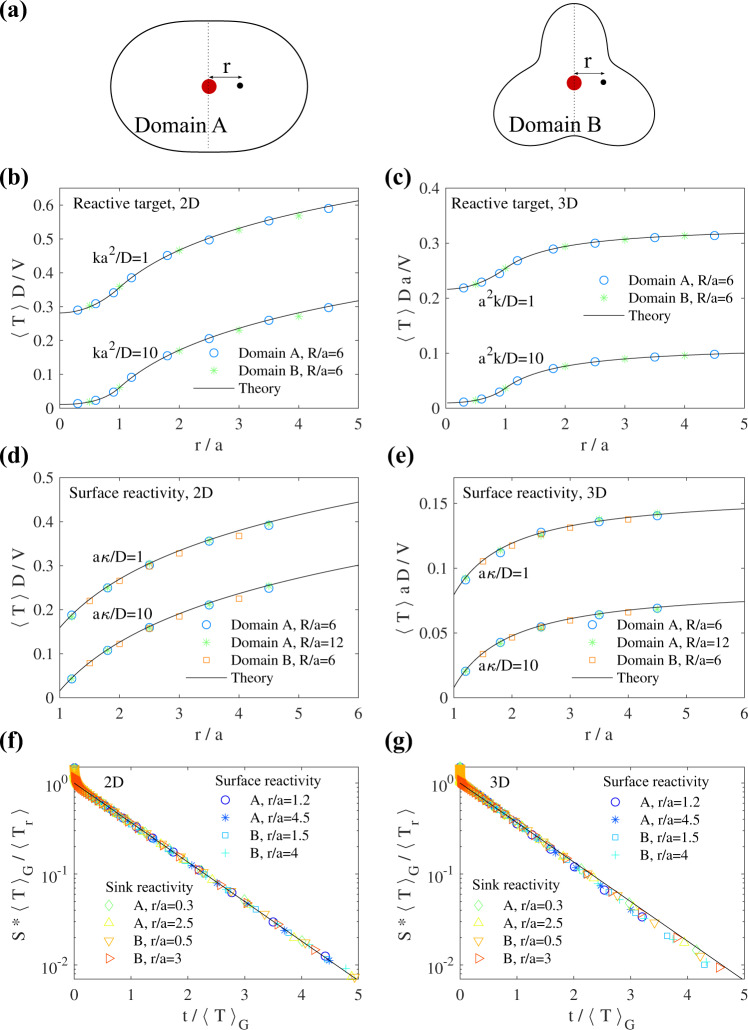
Distribution of reaction times for diffusive processes in various confining domains, for sink and surface reactivity. (**a**) Geometry of the confining domains (called *A* and *B*) that are considered for stochastic simulations. In 2D, these domains are defined in polar coordinates by *r*(*θ*) = *Rf*(*θ*) with $f=1.6(1+0.5{\cos }^{2}\theta )$ for domain *A* and $f=1.6(1+0.1\sin \theta +0.3\sin 3\theta )$ for domain *B*. Domains in 3D are obtained by considering revolution of 2D surfaces around the vertical dashed line. The geometry of the target (red sphere) and initial position of the random walker are indicated. In the figure, we have used *R* = 6*a*. (**b**)–(**e**) Results of stochastic simulations for the mean reaction time in 2D/3D, for surface/sink reactivity, compared to our theoretical expressions. (**f**) and (**g**) Rescaled survival probabilities for 2D/3D simulations, all parameters are in legend except for *R*/*a* = 6 and *ka*^2^/*D* = 1 (for sink reactivity) and *κa*/*D* = 1 (for surface reactivity). In 3D we evaluated 〈*T*〉_G_ = *Vϕ*(*∞*). In 2D, we used 〈*T*〉_G_ = *Vϕ*(1) + 〈*τ*〉_G_ where 〈*τ*〉_G_ was evaluated numerically for each domain. In all simulations we used a time step Δ*t* = 10^−4^*a*^2^/*D*. For surface reactivity we implemented our simulation algorithm by using ref. ^49^. Error bars (95% confidence intervals) are smaller than symbols.

These results for both models (i) and (ii) have been confirmed by numerical simulations for confining volumes of various shapes (see Fig. 3), which have been chosen as representative of nonspherical volumes, displaying anisotropy (*A*) or protrusions (*B*). Importantly, numerical results confirm our prediction that for *d* ≥ 2 the full distribution is still given by Eq. (10) for both continuous models (where the case *d* = 2 is considered as noncompact) [Fig. 3(f–g)].

## Conclusions

We have provided a general formalism to determine the reaction time distribution for imperfect reactions involving the broad class of diffusive and anomalously diffusive Markovian transport processes in confinement. We have investigated several representative mechanisms of imperfect reactivity to test the robustness of our conclusions. Importantly, our results show that the first moment alone, although not representative of typical reaction times, gives access to the full distribution in the large volume limit, which allows to quantify reaction kinetics at all timescales. Thanks to this property, our formalism can be adapted in principle to refined mechanisms of imperfect reactivity (gating, orientational constraints...), as soon as the mean first passage time can be asymptotically determined. Remarkably, and counter-intuitively, we find that in the large volume limit the reaction time distribution is identical to that of the first passage time upon an appropriate rescaling of parameters. This implies that for compact transport processes, the reaction time distribution is broadly distributed with large fluctuations even in the reaction controlled regime where the mean reaction time is independent of the transport process. This is in striking contrast with the naive prediction of exponentially distributed reaction times for first-order kinetics, which in fact is valid only for extremely low reactivity. This unexpected property could lead to large fluctuations of concentrations—as observed in the context of gene expression—even in simple reaction schemes, and even for low reactivity. We expect that the main effect identified here, i.e. that complex first passage properties due to compact transport do not disappear for imperfect reactivity, could be extended to more general processes that are more complex than scale-invariant Markovian processes, and to more complex reactions schemes potentially involving competitive reactions. This will be the subject of future works.

## Supplementary information

Supplementary Information

## Data Availability

The numerical data presented in Figures 2 and 3 are available from the corresponding author on reasonable request.

## Code availability

The code that generated the data presented in Figures 2 and 3 is available from the corresponding author on reasonable request.
