# Supplementary material for: Universal kinetics of imperfect reactions in confinement
Source: Commun Chem. 2021 Nov 11;4:157. doi: 10.1038/s42004-021-00591-2 (PMC9814865; doi:10.1038/s42004-021-00591-2)
Supplement: Supplementary file 1 — Supplementary Information [file 42004_2021_591_MOESM1_ESM.pdf]

Universal kinetics of imperfect reactions in confinement
Supplementary Information

Thomas Guérin, Maxim Dolgushev, Olivier Bénichou, and Raphaël Voituriez

In this Supplementary Information, we provide:

- A brief derivation of Eqs. (2) and (3) in the main text (Supplementary Note 1).
- Details on the derivation of Eq. (7) in the main text, for the calculation of the distribution of reaction times for compact searches (Supplementary Note 2).
- Details on reaction times on fractal networks (Supplementary Note 3), including the method we used to calculate the distribution of first reaction times on large deterministic fractal networks, additional results on Vicsek fractals (Supplementary Figure 1), additional results for other fractals (Supplementary Figure 2), details on simulations on the percolation cluster and a summary table of fractal dimensions for all networks considered in this work (Supplementary Table 1).

Supplementary Note 1: Derivation of Eqs. (2),(3) in the main text

We start from Eq.(1) in the main text:

$$F(T|\mathbf{r},p)=\sum_{n=1}^{\infty}\int_0^{\infty}\mathrm{d}\tau_{\mathbf{r}}\left[\prod_{k=2}^{n}\int_0^{\infty}d\tau_1^{(k)}F_1^*(\tau_1^{(k)})\right]p(1-p)^{n-1}F^*(\tau_{\mathbf{r}}|\mathbf{r})\delta\left(T-\tau_{\mathbf{r}}-\sum_{k=2}^{n}\tau_1^{(k)}\right), \tag{1}$$

Now, taking the Laplace transform with respect to the variable T, we obtain

$$\tilde{F}(s|\mathbf{r},p)=\int_0^{\infty}dTe^{-sT}\sum_{n=1}^{\infty}\int_0^{\infty}\mathrm{d}\tau_{\mathbf{r}}\left[\prod_{k=2}^{n}\int_0^{\infty}d\tau_1^{(k)}F_1^*(\tau_1^{(k)})\right]p(1-p)^{n-1}F^*(\tau_{\mathbf{r}}|\mathbf{r})\delta\left(T-\tau_{\mathbf{r}}-\sum_{k=2}^{n}\tau_1^{(k)}\right), \tag{2}$$

We change the order of integration and integrate with respect to T first:

$$\tilde{F}(s|\mathbf{r},p)=\sum_{n=1}^{\infty}\int_0^{\infty}\mathrm{d}\tau_{\mathbf{r}}\left[\prod_{k=2}^{n}\int_0^{\infty}d\tau_1^{(k)}F_1^*(\tau_1^{(k)})\right]p(1-p)^{n-1}F^*(\tau_{\mathbf{r}}|\mathbf{r})e^{-s\tau_{\mathbf{r}}-s\sum_{k=2}^{n}\tau_1^{(k)}} \tag{3}$$

The integrals factorize, leading to

$$\tilde{F}(s|\mathbf{r},p)=\sum_{n=1}^{\infty}[\tilde{F}_1^*(s)]^{n-1}p(1-p)^{n-1}\tilde{F}^*(s|\mathbf{r}) \tag{4}$$

We recognize a geometrical series and we thus obtain

$$\tilde{F}(s|\mathbf{r},p)=\frac{p\tilde{F}^*(s|\mathbf{r})}{1-\tilde{F}_1^*(s)(1-p)} \tag{5}$$

which is Eq. (2). Now, using the small-s expansions

$$\tilde{F}^*(s|\mathbf{r})=1-s\langle\tau_{\mathbf{r}}\rangle+\mathcal{O}(s^2),\qquad \tilde{F}_1^*(s)=1-s\langle\tau_1\rangle+\mathcal{O}(s^2),\qquad \tilde{F}(s|\mathbf{r},p)=1-s\langle T_{\mathbf{r}}(p)\rangle+\mathcal{O}(s^2), \tag{6}$$

we see that an expansion of Supplementary Equation (4) at linear order in s leads to

$$\langle T_{\mathbf{r}}(p)\rangle=\langle\tau_{\mathbf{r}}\rangle+\frac{1-p}{p}\langle\tau_1\rangle. \tag{7}$$

which is Eq. (3).

Supplementary Note 2: Derivation of Eq. (7): Distribution of first reaction times for compact searches

Here we identify the distribution of reaction times in discrete fractal networks for compact searches. Our strategy is to identify all its moments by exploiting the fact that $\tilde{F}(s|r,p)$ is the generating function of the moments, i.e.

$$\tilde{F}(s|r,p) = \langle e^{-sT_r(p)} \rangle = \sum_{n=0}^{\infty} (-1)^n \ s^n \langle T_r^n(p) \rangle / n! \tag{8}$$

Using the fact that a similar relation can be written for all distributions, we write Eq. (3) (in the main text) by using the expansion of $\tilde{F}^*$ and $\tilde{F}_1^*$ as an infinite series (involving the moments $\langle \tau_r^n \rangle$ and $\langle \tau_1^n \rangle$) and use the expansion of the function $1/(1-x)$ near $x=0$ to obtain

$$\tilde{F}(s|r,p) = \frac{p \ \tilde{F}^*(s|r)}{1-(1-p)\tilde{F}_1^*(s)} = \left[1 + \sum_{q=1}^{\infty} \frac{(-1)^q \ s^q \langle \tau_r^q \rangle}{q!}\right] \times \left\{1 + \sum_{m=1}^{\infty} \left[\sum_{n=1}^{\infty} \frac{(1-p)(-1)^n}{p \ n!} \langle \tau_1^n \rangle s^n\right]^m\right\}. \tag{9}$$

At this stage, identifying all moments $\langle T_r^n(p) \rangle$ (thus the coefficient of s^n for any n in the above expression) seems an intractable task. However, we recall that the moments of the mean first passage time are known [1, 2] to scale as

$$\langle \tau_r^n \rangle \sim a_n r^{d_w - d_f} R^{d_f + (n-1)d_w}, \tag{10}$$

where the coefficients a_n do not depend on the geometry. Similar scaling (with $r=1$), holds for $\langle \tau_1^n \rangle$. These scalings can be used to show that products of moments are negligible compared to moments of higher order, for example the following estimate

$$\frac{1}{p^M} \langle \tau_1^{n_1} \rangle \langle \tau_1^{n_2} \rangle ... \langle \tau_1^{n_M} \rangle \ll \frac{1}{p} \langle \tau_1^{n_1+n_2+...+n_M} \rangle \tag{11}$$

holds when $R \gg l_c$ (for any $M \geq 2$ for which all $n_i \geq 1$); and the following relation is also valid:

$$\langle \tau_r^{n_1} \rangle \langle \tau_1^{n_2} \rangle \underset{r \ll R}{\ll} \langle \tau_r^{n_1+n_2} \rangle. \tag{12}$$

Using Supplementary Equation (11), we see that for all n, the terms coming from the $m=1$ term of the series dominate all the others (because all coefficients of s^n generated by products of moments are negligible compared to the corresponding term coming from $m=1$ in the series). This leads to

$$\tilde{F}(s|r,p) \simeq \left[1 + \sum_{q=1}^{\infty} \frac{(-1)^q \ s^q \langle \tau_r^q \rangle}{q!}\right] \times \left\{1 + \sum_{n=1}^{\infty} \frac{(1-p)(-1)^n}{p \ n!} \langle \tau_1^n \rangle s^n\right\}. \tag{13}$$

Next, using Supplementary Equation (12) we see that all products of moments are negligible compared to terms that are not products of moments, so that

$$\tilde{F}(s|r,p) \simeq 1 + \sum_{q=1}^{\infty} \frac{(-1)^q \langle \tau_r^q \rangle}{q!} s^q + \sum_{n=1}^{\infty} \frac{(1-p)(-1)^n}{p \ n!} \langle \tau_1^n \rangle s^n. \tag{14}$$

This means that for all $q \geq 1$

$$\langle T_r^n(p) \rangle \underset{R \to \infty}{\sim} \langle \tau_r^n \rangle + \frac{(1-p)}{p} \langle \tau_1^n \rangle. \tag{15}$$

Up to now, the only approximation is the large volume limit. Let us do another approximation: we assume that that the scaling Supplementary Equation (10) holds also for $r=1$; such approximation has proved accurate for the first moment [3]. In this case we get

$$\langle T_r^n(p) \rangle \simeq a_n \left[r^{d_w - d_f} + \frac{(1-p)}{p}\right] R^{d_f} R^{d_w(n-1)}. \tag{16}$$

We realize that the above relation can also be written as

$$\langle T_r^n(p) \rangle \simeq \frac{\langle T_r(p) \rangle}{\langle \tau_r \rangle} \langle \tau_r^n \rangle, \tag{17}$$

which means that all moments of the reaction time are proportional to the moments of the first passage time with a proportionality factor which does not depend on n. This implies a proportionality between the two distributions:

$$F(t|r,p) \simeq \frac{\langle T_r(p)\rangle}{\langle \tau_r\rangle} F^*(t|r). \tag{18}$$

Using the FPT distribution given in Ref. [1] finally leads to Eq. (7) in the main text. Note finally that Supplementary Equation (17) holds in both limits of strong and weak reactivity, so that the hypothesis Supplementary Equation (10) is required only in the crossover regime.

Supplementary Note 3: Additional details on the distribution of reaction times on fractal networks

1. *Details on the method to obtain reaction time distribution on large deterministic fractal networks*

We consider the dynamics of a random walker on a network of N sites and connectivity matrix M, such that $M_{ij} = -1$ when sites i and j are connected and the diagonal elements are $M_{ii} = f_i$, with f_i the functionality, or connectivity of site i (i.e. the number of linked neighbors). Let us consider a random walker on this network, and let us call $p_i(t)$ be the probability to find it at time t at site i. We assume that, between t and $t+dt$, there is a probability $\mu\ dt$ (for each edge) to jump on this edge, so that the average waiting time on site i is $(f_i\mu)^{-1}$). If we consider also one reactive site i_0, which is absorbing with rate k (imperfect reactivity), the master equation for this dynamics is

$$\partial_t \mathbf{p} = -M\mathbf{p} - k\ \mathbf{u}\ ({}^t\mathbf{u}\cdot\mathbf{p}). \tag{19}$$

where $\mathbf{u}$ is a column vector which encodes the position of the reactive site ($u_i = 0$ for all i except for $u_{i_0} = 1$), and $\mathbf{p} = {}^t(p_1, p_2...)$. Note that we have chosen the units of time so that $\mu = 1$. With this dynamics, the stationary probability in absence of reaction is $p_i = 1/N$ so that there is no confusion between averages over stationary configurations and uniform averages. M is symmetric so that it can be expressed as $M = QDQ^{-1}$ with Q an orthonormal matrix ($Q^{-1} = {}^tQ$) and D a diagonal matrix of positive eigenvalues $0 = \lambda_1 < \lambda_2 < ... < \lambda_N$ (possibly degenerate). We pose

$$\mathbf{q} = Q^{-1}\mathbf{p}, \qquad \mathbf{v} = Q^{-1}\mathbf{u}. \tag{20}$$

The dynamics in the space of eigenmodes reads

$$\partial_t \mathbf{q} = -D\mathbf{q} - k\mathbf{v}\ ({}^t\mathbf{v}\cdot\mathbf{q}). \tag{21}$$

Now, the distribution of first reaction times is simply $F(t) = k\ ({}^t\mathbf{u}\cdot\mathbf{p}) = k\ ({}^t\mathbf{v}\cdot\mathbf{q})$. Taking the Laplace transform of Supplementary Equation (21) leads (after a few manipulations) to

$$\tilde{F}(s) = \frac{1}{1 + \frac{k}{sN} + k\sum_{\lambda\neq 0}\frac{1}{s+\lambda}\sum_{i\in I(\lambda)} v_i^2}\left[\frac{k}{sN} + k\sum_{\text{distinct }\lambda\neq 0}\frac{1}{s+\lambda}\sum_{i\in I(\lambda)} v_i q_i(0)\right], \tag{22}$$

where we have decomposed the sum over distinct values of λ different from zero, and $I(\lambda)$ represents the ensemble of indexes i so that $\lambda_i = \lambda$. Now, the key point is that $\sum_{i\in I(\lambda)} v_i^2$ is actually the squared norm of the projection $P_\lambda\mathbf{u}$ over the eigensubspace associated to λ. Similarly, $\sum_{i\in I(\lambda)} v_i q_i(0)$ is the scalar product between $P_\lambda\mathbf{u}$ and the projection $P_\lambda\mathbf{p}(t=0)$ of the vector of initial probabilities over the eigensubspace associated to λ. Although brute force diagonalization of the connectivity matrix M is in practice limited to a few thousands sites N, we can implement a procedure to identify iteratively (from generation g to $g+1$) all the eigensubspaces. We refer to Ref. [4] for details of such iterative procedures for (i) the case of Vicsek fractals and (ii) the dual Sierpinski gasket. In practice, these projections can be computed relatively cheaply (within a few days on a single processor) up to $g = 13$ (dual Sierpinski gasket, so that $N \simeq 1.6\times 10^6$ sites), and for Vicsek fractals $g = 8, 7, 6$ for global functionalities $f = 3, 4, 6$, respectively.

Note that calculating these projections for a given vector $\mathbf{u}$ and $\mathbf{p}(t=0)$ thus gives access to the whole probability distribution in Laplace space, thus for all times after numerical Laplace inversion, for all values of the reactivity parameter k. In particular the first moments can be expressed as

$$\langle T\rangle = \frac{N}{k} + \sum_{\text{distinct }\lambda\neq 0}\frac{N}{\lambda}\sum_{i\in I(\lambda)}[v_i^2 - v_i q_i(0)]. \tag{23}$$

Note that the global mean reaction time is available by choosing the stationary distribution for $p_i(t=0) = 1/N$. Finally we note that the probability p to be absorbed at each visit of the target in this model is

$$p = \int_0^\infty dt\ k\ e^{-(k+f_{i_0})t} = \frac{k}{k+f_{i_0}}. \tag{24}$$

2. *Results for Vicsek fractals of different functionalities*

For Vicsek fractals, Different values of d_w and d_f can be explored by varying the functionality of the central bead. In the main text, we show the results for $f = 6$, additional examples are shown on Supplementary Figure 1 and show that our theory is confirmed for different values of f.

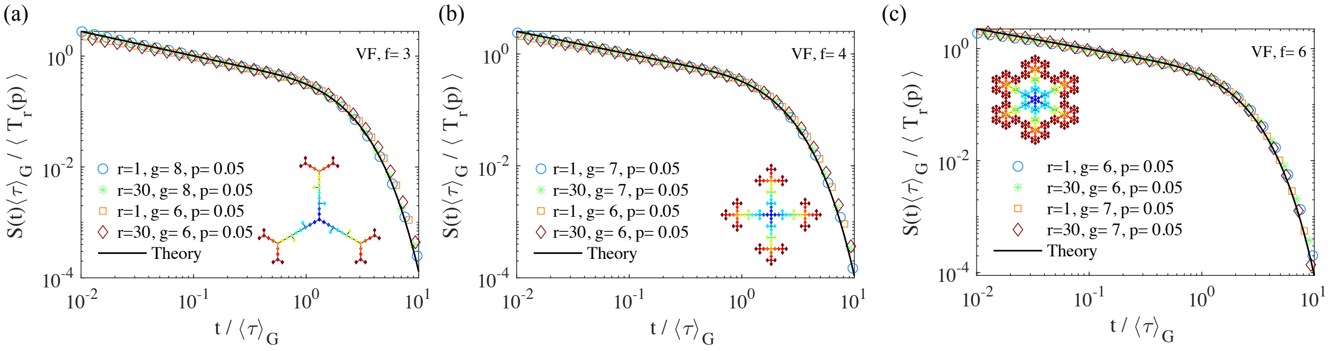

Supplementary Figure 1: **Additional results obtained with the numerical method described in Supplementary Note 3.1 for Vicsek fractals of different functionalities.** (a): $f = 3$, (b): $f = 4$; (c) $f = 6$. In each graph, the network is represented in inset at generation $g = 3$.

3. *Results of stochastic simulations for other fractal networks*

In the main text, we have considered a dynamics on networks for which the average waiting time on each site is inversely proportional to its number of neighbors. Here we consider "traditional" random walk simulations, in which the waiting time at each site is uniform and taken as unity. We have performed stochastic simulations for this dynamics and we have checked that this different type of dynamics does not change the validity of our results. The figure below demonstrates that, once properly rescaled by appropriate first moments, the shape of the survival probability falls into the universality classes predicted in the main text.

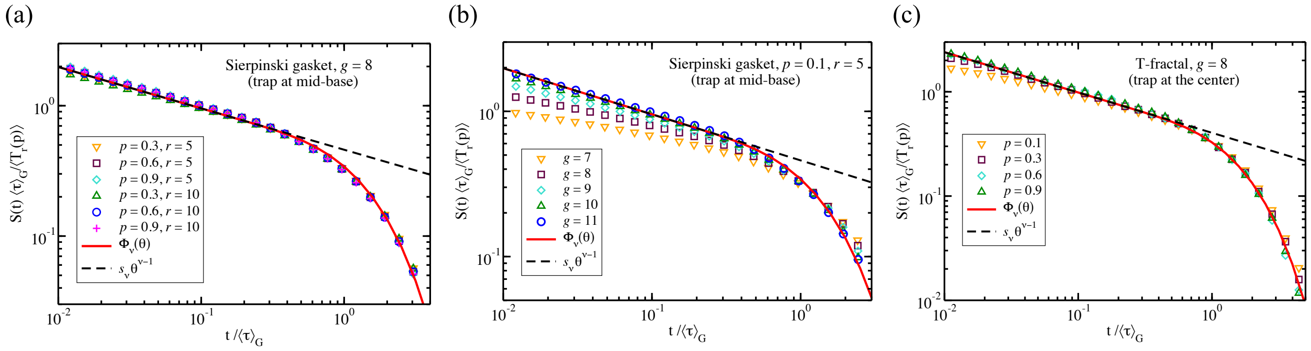

Supplementary Figure 2: **Rescaled survival probability for random walks on fractals.** (a) and (b): Sierpinski gasket (for which $\nu = d_f/d_w = \ln 3/\ln 5$); (c) T-fractal ($\nu = \ln 3/\ln 6$). The red line represents $\Phi_\nu(\theta)$ given in Eq. (8) of the main text, whose algebraic decay (dashed line) contains the prefactor $s_\nu = \left(\frac{2\nu}{1-\nu^2}\right)^\nu \frac{\nu}{\Gamma(1-\nu)}$. For both fractals the global mean first passage time $\langle\tau\rangle_G$ used for rescaling of time [see Eq. (7) of the main text] is known analytically [5, 6]; for T-fractal the simulation data is averaged over chemical distances $r \in [1, 32]$. For the graph in the middle, $p = 0.1$ is fixed, note the convergence towards the predicted value when the size of the system increases (g is the generation number). In all graphs, 95% confidence intervals are smaller than symbol sizes.

Supplementary Table 1: Summary of fractal dimensions for all fractal networks considered in this work

Fractal	Spatial dimension (d_f)	Walk dimension (d_w)
Vicsek fractal (f=3)	$\ln(1+3)/\ln 3 \simeq 1.26$	$1 + d_f \simeq 2.26$
Vicsek fractal (f=4)	$\ln(1+4)/\ln 3 \simeq 1.47$	$1 + d_f \simeq 2.47$
Vicsek fractal (f=6)	$\ln(1+6)/\ln 3 \simeq 1.77$	$1 + d_f \simeq 2.77$
Sierpinski Gasket	$\ln 3/\ln 2 \simeq 1.59$	$\ln 5/\ln 2 \simeq 2.32$
Dual Sierpinski Gasket (DSG)	$\ln 3/\ln 2 \simeq 1.59$	$\ln 5/\ln 2 \simeq 2.32$
T-fractal	$\ln 3/\ln 2 \simeq 1.59$	$\ln 6/\ln 2 \simeq 2.59$
2D (bond) Percolation cluster	$91/48 \simeq 1.90$	2.878

4. Details on simulations for random walks on the percolation cluster

To generate a percolation cluster, we have used a regular 200×200 periodic two-dimensional square lattice on which we have removed randomly half of the bonds. Then, we have identified the connected network of maximal size (with the algorithm of Ref. [7]) on which random walks simulations were performed, by prescribing that the waiting time at each site is inversely proportional to its connectivity, so that the uniform distribution is also the equilibrium distribution. For each run, the target and the initial position were chosen uniformly with the constraint of fixed (chemical) distance between them. To generate Fig. 2(e) of the main text, the MRT was estimated from Eq. (5), and the GMFPT was identified numerically for the percolation cluster under consideration.

Supplementary References

[1] Bénichou, O., Chevalier, C., Klafter, J., Meyer, B. & Voituriez, R. Geometry-controlled kinetics. *Nat. Chem.* **2**, 472–477 (2010).

[2] Levernier, N., Bénichou, O., Guérin, T. & Voituriez, R. Universal first-passage statistics in aging media. *Phys. Rev. E* **98**, 022125 (2018).

[3] Bénichou, O., Meyer, B., Tejedor, V. & Voituriez, R. Zero constant formula for first-passage observables in bounded domains. *Phys. Rev. Lett.* **101**, 130601 (2008).

[4] Dolgushev, M., Guérin, T., Blumen, A., Bénichou, O. & Voituriez, R. Contact kinetics in fractal macromolecules. *Phys. Rev. Lett.* **115**, 208301 (2015).

[5] Agliari, E. Exact mean first-passage time on the t-graph. *Phys. Rev. E* **77**, 011128 (2008).

[6] Haynes, C. P. & Roberts, A. P. Global first-passage times of fractal lattices. *Phys. Rev. E* **78**, 041111 (2008).

[7] Newman, M. E. J. & Ziff, R. M. Fast monte carlo algorithm for site or bond percolation. *Phys. Rev. E* **64**, 016706 (2001).
